# Changes in Sensory Properties, Physico-Chemical Characteristics, and Aromas of Ras Cheese under Different Coating Techniques

**DOI:** 10.3390/foods12102023

**Published:** 2023-05-17

**Authors:** Dina A. Amer, Abdinn A. M. Albadri, Hanaa A. El-Hamshary, Yasser Nehela, Abeer H. Makhlouf, Mohamed Y. El-Hawary, Sameh A. Awad

**Affiliations:** 1 Department of Food Science and Technology, Faculty of Agriculture, Tanta University, Tanta 31527, Egypt; 2 Department of Biology, College of Science, King Khalid University, Abha 62529, Saudi Arabia; abdin@kku.edu.sa; 3 Department of Agricultural Botany, Faculty of Agriculture, Tanta University, Tanta 31527, Egypt; 4 Department of Plant Pathology, Citrus Research and Education Center, University of Florida, Lake Alfred, FL 33850, USA; 5 Department of Agricultural Botany, Faculty of Agriculture, Minufiya University, Shibin El-Kom 32511, Egypt; 6 Dairy Microorganisms and Cheese Research Laboratory (DMCR), Department of Dairy Science and Technology, Faculty of Agriculture, Alexandria University, Alexandria 21545, Egypt; sameh.awad@alexu.edu.eg

**Keywords:** dairy, milk, Ras cheese, coating, natamycin, volatiles, VOCs, GC-MS, paraffin wax, aroma

## Abstract

Ras cheese is one of the main hard cheeses in Egypt and is well-known worldwide. Herein, we investigated the potential effects of different coating techniques on the physico-chemical characteristics, sensory properties, and aroma-related volatile organic compounds (VOCs) of Ras cheese over a six-month ripening period. Four coating techniques were tested, including (I) uncoated Ras cheese (the benchmark control), (II) Ras cheese coated with paraffin wax (T1), (III) Ras cheese coated with a plastic film under a vacuum (PFUV; T2), and (IV) Ras cheese coated with a plastic film treated with natamycin (T3). Although none of the treatments significantly affected the salt content, Ras cheese coated with a plastic film treated with natamycin (T3) slightly reduced the moisture content over the ripening period. Moreover, our findings revealed that while T3 had the highest ash content, it showed the same positive correlation profiles of fat content, total nitrogen, and acidity % as the control cheese sample, indicating no significant effect on the physico-chemical characteristics of the coated cheese. Furthermore, there were significant differences in the composition of VOCs among all tested treatments. The control cheese sample had the lowest percentage of other VOCs. T1 cheese, coated with paraffin wax, had the highest percentage of other volatile compounds. T2 and T3 were quite similar in their VOC profiles. According to our GC-MS findings, thirty-five VOCs were identified in Ras cheese treatments after six months of ripening, including twenty-three fatty acids, six esters, three alcohols, and three other compounds identified in most treatments. T2 cheese had the highest fatty acid % and T3 cheese had the highest ester %. The development of volatile compounds was affected by the coating material and the ripening period of the cheeses, which played a major role in the quantity and quality of volatile compounds.

## 1. Introduction

Ras cheese is the main traditional and the most popular hard cheese in Egypt. It is close to the Greek variety Kefalotyri. Additionally, It belongs to the same family as Pecorino Romano and Manchego cheese [1]. It is made from raw cow’s milk or a mixture of cow’s and buffalo milk without using starter cultures [2]. However, to the best of our knowledge, differences(s) in sensory properties, physico-chemical characteristics, and aromas of Ras cheese and other types of cheese are poorly studied. Briefly, the unique characteristics of Ras cheese compared with other cheese types might be due to its traditional manufacturing method depending on the fermentation, which occurs by the native microflora from the raw milk and the environment. Ras cheese is usually stored in humid and uncontrolled hygienic conditions in not hermetically closed rooms without a coating. Collectively, these maturation conditions support the growth of molds and yeasts. Consequently, the final flavor and texture will be influenced by the actions of all these factors. [3]. The traditional Ras cheese usually had some bad features that resulted in mis-achievement of the Egyptian standard, such as containing high levels of yeast, mold, and other undesirable microorganisms [4]. The bad quality of traditional Ras cheese being susceptible contamination with yeasts and molds caused potential problems in its quality, science ripening period, storage, or marketing [5].

Recently, flavor and aroma analysis of hard cheese has been the subject of considerable research. The aroma of hard cheese is controlled by the presence of several volatile organic compounds (VOCs) and their compositions that determine the specific aroma of produced cheese. The odor threshold value (OTV) is a main component of aroma analysis of hard cheese and can serve as a useful warning property. The odor threshold is the minimum concentration of a substance at which most panelists can detect and identify the characteristic odor of a substance. However, the OTV must be used cautiously because olfactory perception varies among individual panelists.

Cheese manufacturing was modified using pasteurized milk, application of the cheese coating, and the modification of the salting techniques to be sweet, which could be utilized in other applications and be ripened under controlled conditions [6]. These modifications of Ras cheese in both manufacturing, as well as ripening, must not have a side effect on the physico-chemical properties and acceptability of the final cheese.

One of the major losses throughout the storage and ripening period is commercialization, which occurs because of the contamination of cheese with microorganisms. Additionally, the emergence of off-flavor decreases the quality of the uncoated stored cheese. The loss in moisture content in stored cheese could be related to increasing its hardness, as well as leading to unacceptable sensorial characteristics [7]. The coating is one of the messianic processes in food and cheese manufacturing, as well as commercialization. Coating cheese helps to protect and maintain the safety, quality, and shelf-life of cheese during the storage period [8]. The characteristics of cheese coating materials are very important, such as water vapor, a gas barrier, and the size and shape of the coating materials to ensure the safety and quality of the coated cheese [9]. The process of coating materials or technique must be regarded because cheese is a complex biological dynamic matrix that has various physical, chemical, and microbial changes that happen throughout the storage or ripening period [10].

The usage of a plastic film as a coating material for ripened cheese could have good properties due to its excellent barrier characteristics against moisture and oil losses, as well as its good properties versus corrosion; it is also cheap. All these properties made it highly convenient for use [11]. The coating of cheese could be performed during many phases of manufacturing such as before ripening, during the ripening process, or even after ripening finished. The coating of cheese during ripening may occur due to regulating the moisture content in cheese and protecting cheese from microorganisms. The materials used in coating the cheese could be a film from different things such as a layer of polyvinyl lactate, paraffin wax, or plastic under a vacuum, as well as an antimicrobial substance, such as natamycin.

A vacuum coating could reduce oxidative destruction, as well as hinder aerobic microorganisms, which could lead to extending the shelf-life of stored cheese. In the vacuum coating technique, common plastic material is used, which forbids the dehydration and loss of weight in cheese throughout the ripening period. It was also useful in the absorption of unacceptable flavors from external sources [12]. The vacuum coating technique could be more interesting for hard cheese and semi-hard cheese, which are ripened by bacteria at a slow rate because of their low content of moisture and salt. Waxes that are used in cheese coating are a heterogeneous class of lipids that contain hydrocarbons, as well as other non-polar substances. The material of natural waxes could be from vegetables, animals, or even minerals. Waxes could be used in coatings for hard or semi-hard cheese varieties [13,14].

Waxes that are used in cheese coating have different functions, such as protecting cheeses from a loss of water by evaporation, which affects the weight of mature cheese and prevents mold growth throughout the storage period and transportation [14]. Many traditional cheese manufacturers use waxes, mainly paraffin wax in cheese coatings [15,16]. Waxes that are used in cheese coatings should have a low tendency to cleft since imposing outside forces should have low water vapor presence. Additionally, they should have the generally recognized as safe (GRAS) status, as well as a low effect on the environment, to prevent defects in cheese coating [17].

Coating cheese with films that had antimicrobial substances are also known as active coatings. This technique of coating allows the antimicrobial substance to ensure the safety of coated foods [18]. Natamycin is known as a fungicide that belongs to polyethylene antibiotics and is produced by the aerobic fermentation of *Streptomyces natalensis*, as well as related species [19]. It is commonly used in food preservation, especially in dairy products such as cheese to prevent microorganisms’ contamination. It was also approved by the Food and Drug Administration and the European Community as a safe natural preservative in foods [20,21].

Some factors adjust the efficiency of natamycin’s application on the food surface. Likewise, inadequate water solubility accelerated migration and inactivation by food matrix components [22,23]. Natamycin is used at different concentrations from 1 to 20 ppm. Natamycin is granted as an anti-mycotic in surface and cheese treatments. It is permitted for surface treatment of hard, semi-hard, and semi-soft cheeses. Nutrient sources of foods and the EFSA Panel on Food Additives have illustrated that natamycin is poorly absorbed; there was an adequate margin of safety and no concern for the induction of antimicrobial resistance related to its applications [24].

Therefore, this investigation was carried out to investigate the possibility of using different materials such as paraffin wax, the plastic film under a vacuum (PFUV), and the plastic film treated with natamycin in the coating of Ras cheese during ripening and its effect on the quality characteristics of the final cheese.

## 2. Materials and Methods

### 2.1. Materials

Fresh full-fat cow’s milk was obtained from the farm of the Faculty of Agriculture, Alexandria University. The composition of cow’s milk was as follows: fat 3.6%, protein 3.24%, T.S. 13.40%, acidity 0.18%, and pH 6.68. Starter cultures freeze-dried lactic culture (YF-L904) for direct vat set (DVS) consists of *Streptococcus thermophilus* and *Lactobacillus delbreuckii* subsp. *Bulgaricus* was obtained from Chr. Hansen Lab., Denmark. Liquid rennet was obtained from the local market, Alexandria. Annatto, sodium chloride, calcium chloride, and paraffin wax were obtained from EL-Nasr Company, Alexandria. The plastic film (polyethylene food-safe plastic) and the plastic film treated with natamycin were also obtained from EL-Nasr Company, Alexandria. A vacuum packaging machine, WAAGE. A Bilance Elettroniche, WAAGE s.r.i. with Casilina Nord 81049 S, Pietro Infine (CE) Italy, was obtained from the Faculty of Agriculture, Alexandria University.

### 2.2. Ras Cheese Manufacture

The control Ras cheese, which was used as a benchmark cheese and other cheese treatments, was made in the dairy pilot plant, the Department of Dairy Science and Technology, Alexandria University. The raw cow’s milk was pasteurized at 63 °C for 30 min and cooled to 38 °C and then transferred to a cheese vat. The control Ras cheese was made according to Hofi et al. [25], while modifications in the salting process of Ras cheese were made according to Awad [26]. All cheeses were salted by brine solution (24%) for 24 h without dry salting and then treated as follows: uncoated Ras cheese (benchmark control), Ras cheese coated with paraffin wax (T1), Ras cheese coated with a plastic film under a vacuum (PFUV; T2), and Ras cheese coated with a plastic film treated with natamycin. The cheeses were allowed to ripen under the dominant condition of a temperature of 13 ± 2 °C and a relative humidity of about 85%. Cheese treatments were sampled and analyzed at 1, 30, 60, 120, and 180 days of ripening.

### 2.3. Physico-Chemical Analysis of Ras Cheese

Cheese samples were analyzed according to the A.O.A.C. methods [27]. The moisture content was determined according to British Standard Institution. The titratable acidity and pH value of the cheese samples were measured in a slurry prepared by macerating 20 g of grated cheese in 20 mL of deionized water using a pH meter (Mi 151 PH, ORP/Temperature Bench Meter). Titratable acidity was measured by recording the amount of the sodium hydroxide solution (0.1 N NaOH) that was used to reach the endpoint. The fat % of the cheeses was determined using Gerber’s method, and total nitrogen (%) was determined using the Kjeldahl method and semi-atomized according to the A.O.A.C. methods [27]. The ash content was determined using muffle furnaces at 550 °C. The salt content was determined by titration methods using silver nitrate (AgNO_3_) 0.05 N and potassium chromate (2%), according to Pearson’s method [28]. Next, 0.5 g of the cheese sample was then added to 50 mL of distilled water and was then boiled directly over the fire. We filtered it and then washed the precipitate twice. We completed the filtration with 100 mL of water, 5 mL of filtrate, and added 1 mL of potassium chromate (2%), then dripped AgNO_3_ (0.05 N) until a stable red color appeared, and then we recorded the values of AgNO_3_.

### 2.4. Sensory Evaluation

The sensory qualities of the cheese samples were assessed after 6 months of the ripening period with a regular scoring panel of twelve trained panelists from the Dairy Science and Technology Department at Alexandria University. The panelists were surveyed for flavor and texture [3]. The scale was as follows: 1, inadequate; 2, enough; 3, acceptable; and 4, very good. The texture was graded on a scale of 1 (soft), 2 (average), 3 (hard), and 4 (extremely hard). The panelists were asked to assign the samples an overall grade of 1–100.

### 2.5. Analysis of Volatile Organic Compounds (VOCs)

Volatile compounds of the cheese samples were identified using purge-and–trap thermal desorption cold-trap (TDCT) gas chromatography–mass spectrometry (GC-MS) [29]. As follows, we prepared 20 mL of cheese slurry in double-distilled water (1:2 *w*/*v*). The cheese samples were purged with 150 mL min^−1^ of helium gas for 30 min at 42 °C and volatile components were trapped on an absorbent trap containing a carbon trap (80 mg, 20–40 mesh, Supelco, Sigma-Aldrich, St. Louis, MO, USA). The trapped compounds were transferred onto a capillary column of a gas chromatograph using the Chrompack PT1 injector by heating the trap oven at an initial temp of 50 °C for 4 min, ramp 6 °C/min to 150 °C, hold for 2 min, ramp 6 °C/min to 200 °C, hold for 0 min, ramp 6 °C/min to 280 °C, hold for 2 min, Inj = 280 °C, volume = 1 µL, Spblit = 20:1, carrier gas = He, solvent delay = 5.00 min, transfer temp = 280 °C, and source temp = 200 °C. The conditions for the chromatographic separation and mass spectrometry have been conducted according to Engels et al. [29]. Volatile compound composition was assigned spectrum explanation, the comparison of the spectra with bibliographic data, and the comparison of retention times with those of reference compounds, using GC-MS (Model, Clarus 580/560 S, Perkin Elmer, Waltham, MA, USA). Scan 50 to 620Da, column (Elite-5 MS, 30 m 0.25 mm ID 0.25 um df).

### 2.6. Statistical Analysis

The experiment was carried out using a completely randomized design (CRD) with three biological replicates per treatment. All data were statistically analyzed using the analysis of variance (ANOVA) followed by Tukey’s honestly significant difference (HSD) test as a post-hoc analysis to compare between means (*p* < 0.05). Simple linear regression (SLR) was used to better understand the relationship between the studied parameters and the ripening period of the cheese. Moreover, a principal component analysis (PCA) and a two-way cluster analysis were used to analyze the VOC data [30].

## 3. Results

**Figure 1:**
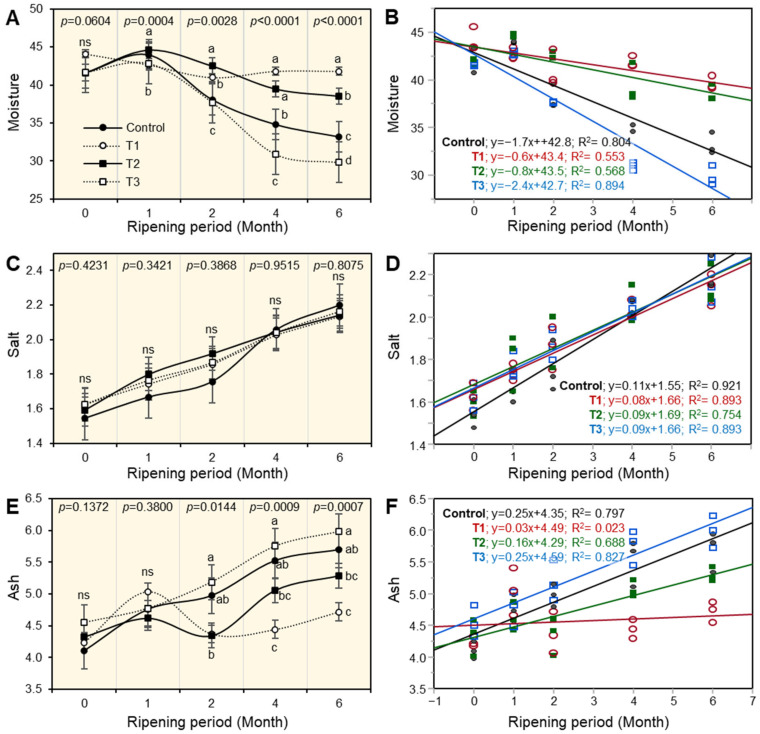
The effect of different coating materials on the physical properties of Ras cheese. (**A**) Moisture content, (**B**) simple linear regression (SLR) between moisture and ripening periods, (**C**) salt %, (**D**) SLR between salt % and ripening periods, (**E**) ash, and (**F**) SLR between ash and ripening periods. Values represent the means ± standard deviation (means ± SD) of three biological replicates (*n* = 3). Different letters indicate statistically significant differences between treatments (*p* < 0.05).

**Figure 2:**
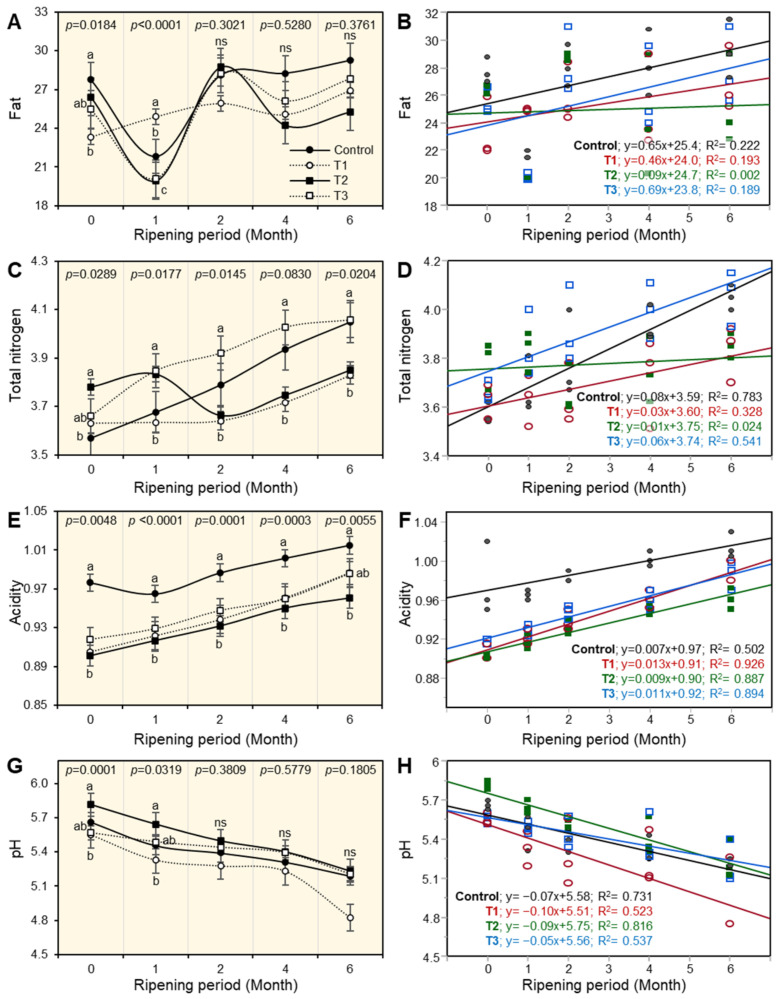
The effect of different coating materials on the chemical and physical properties of Ras cheese. (**A**) Fat, (**B**) simple linear regression (SLR) between fat and ripening periods, (**C**) total nitrogen, (**D**) SLR between total nitrogen and ripening periods, (**E**) acidity, (**F**) SLR between acidity and ripening periods, (**G**) pH values, and (**H**) SLR between pH values and ripening periods. Values represent the means ± standard deviation (means ± SD) of three biological replicates (*n* = 3). Different letters indicate statistically significant differences between treatments (*p* < 0.05).

**Figure 3:**
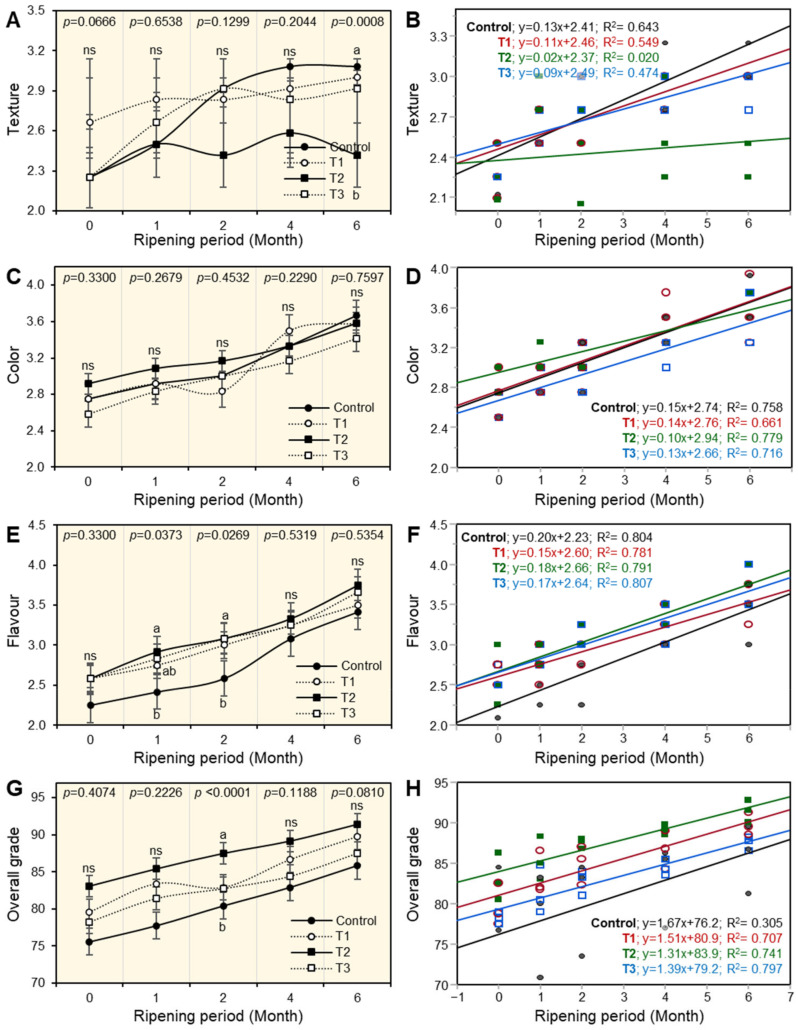
The effect of different coating materials on sensory properties of Ras cheese. (**A**) Texture, (**B**) simple linear regression (SLR) between texture and ripening periods, (**C**) color, (**D**) SLR between color and ripening periods, (**E**) flavor, (**F**) SLR between flavor and ripening periods, (**G**) overall grade, (**H**) SLR between overall grade and ripening periods. Values represent the means ± standard deviation (means ± SD) of three biological replicates (*n* = 3). Different letters indicate statistically significant differences between treatments (*p* < 0.05).

**Figure 4:**
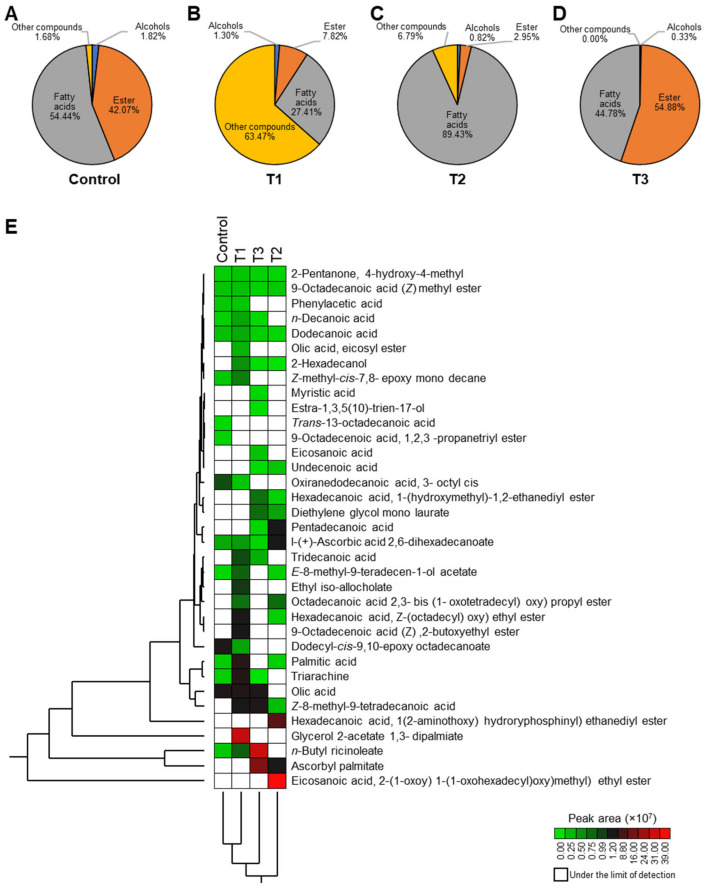
Percentages of different volatile organic compound (VOC) groups of Ras cheese treated with different coating materials over a six-month ripening period. (**A**–**D**) Percentages of different VOC groups of the control, T1-, T2-, and T3-treated Ras cheese, respectively. (**E**) Two-way hierarchical cluster analysis (HCA) and heatmap using the abundance of volatile organic compounds (VOCs) of Ras cheese treated with different coating materials over the six-month ripening period. Rows represent VOC abundance and columns represent treatments. Cells are colored based on abundance. Red represents high abundance and blue represents low abundance.

**Figure 5:**
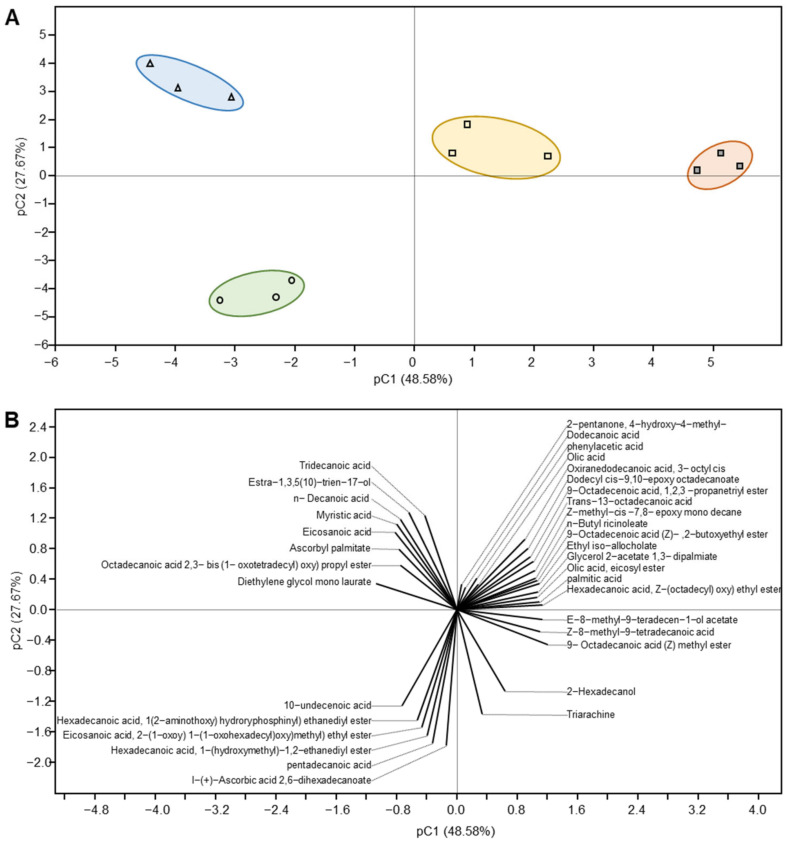
Principal component analysis (PCA) and its associated biplot of volatile organic compounds (VOCs) of Ras cheese treated with different coating materials over a six-month ripening period. (**A**) PCA scatterplot using the abundance of all volatiles and a PCA biplot (**B**).

Changes in the physico-chemical properties of the experimental Ras cheese as affected by different coating materials are shown in Figure 1, Figure 2, Figure 3, Figure 4 and Figure 5. The basic composition of Ras cheese was determined in samples over 6 months.

### 3.1. Moisture Content of Ras Cheese

Changes in the moisture content of Ras cheese as affected by different coating materials are illustrated in Figure 1A. There were significant differences among all cheese treatments from 1 month to 6 months of the ripening period (*p* ˂ 0.05). T1 and T3, coated with paraffin wax and the plastic film treated with natamycin, respectively, had the lowest moisture content compared with the benchmark control sample of cheese and T2 cheese after the first month of the ripening period. By the end of the ripening period, T3 cheese had the lowest moisture content while T1 cheese had the highest content. As observed during the ripening period, moisture content decreased in all cheese treatments; this trend was much more pronounced in the benchmark control sample of cheese and T3 cheese, which had the lowest moisture content at the end of the ripening period.

The leaner regression (SLR), as presented in Figure 1B, was used to better understand the relationship between moisture content and the ripening period of the cheese. Results illustrated that the relationship between moisture content and the ripening period was negatively correlated. The moisture content of all cheeses decreased as the ripening period progressed. Ras cheese treatments, T1 and T2, had strong negative correlations, as illustrated in the following equations: T1: y = −0.6x + 43.4 and T2: y = −0.8x + 43.8. However, the control cheese sample and T3 cheese had a weak negative correlation, as shown in the following equations: C: y = −1.7x + 42.8 and T3: y = 2.4x + 42.7.

### 3.2. Salt Content of Ras Cheese

The salt content can indicate accessibility, as well as the shelf-life of ripened cheese. According to the results in Figure 1C, there were no significant differences in the salt content during the ripening period among all Ras cheese treatments. Furthermore, salt content increased as the ripening period progressed in all treatments.

The results in Figure 1D show the relationship between the salt percentage and Ras cheese during the ripening period using simple linear regression (SLR). There was a strong positive correlation between Ras cheese salt percentage and ripening period progress; this trend was cleared in all Ras cheese experiments, as seen in the following equations: C; y = 0.11x + 1.55; T1; y = 0.08x + 1.66; T2; y = 0.09x + 1.69, and T3; y = 0 > 0.09x + 1.66.

### 3.3. Ash Content of Ras Cheese

There were significant differences (*p* ˂ 0.05) in the ash content between all Ras cheese treatments after two months of the ripening period (Figure 1E). T3 cheese, which was coated with a plastic film treated with natamycin, had the highest ash content compared with other Ras cheese treatments from after two months until the end of the ripening period. Furthermore, the results illustrate the progress of the ripening period, which increased the ash content of Ras cheese treatments.

To better understand the relationship between the ash content and the ripening period, simple leaner regression (SLP) was used (Figure 1F). The results showed that there was a strong correlation between ash content and the progression of the ripening period in all Ras cheese treatments, while T3 cheese had the highest positive correlation, as provided by the following equations: C, y = 0.25x + 4.35; T1, y = 0.03x + 4.49; T2, y = 0.16x + 4.29; and T3, y = 0.25x + 4.59.

### 3.4. Fat Content of Ras Cheese

There were significant differences in the fat content in Ras cheese treatments, as observed in (Figure 2A). The fresh control benchmark sample cheese had a high fat percentage, whereas T1 cheese coated by a plastic film under a vacuum (PFUV) had the highest fat percentage. Additionally, data revealed that T2 and T3 cheese had the lowest and T1 cheese had the highest fat percentage after the first month of the ripening period. The fat percentage in all Ras cheese treatments increased as the ripening period progressed. There were no significant differences in the cheese at the end of the storage period.

To illustrate the relationship between the fat percentage and the ripening period, simple leaner regression (SLR) was used (Figure 2B). The fat percentage and ripening period had a positive correlation; this trend was much more pronounced in the control benchmark cheese and T1 cheese, which had weak positive correlations. Additionally, the results showed that T2 cheese did not correlate with the ripening period, as illustrated by the following equation: T2, y = 0.09x + 23.8.

### 3.5. Total Nitrogen Content of Ras Cheese

The results showed that there were significant (*p* ˂ 0.05) differences in total nitrogen percent in the fresh cheese among all treatments (Figure 2C). The control cheese sample had the highest total nitrogen percentage compared with the other Ras cheese. It could be observed that T2 coated with PFUV cheese had the highest total nitrogen percentage. Throughout other ripening periods, there were no significant differences among all Ras cheese treatments.

Using a simple linear correlation, the results shown in Figure 1D provided a better understanding of the relationship between the total nitrogen percentage and the ripening period. Positive regression was observed in all Ras cheese treatments, but it was more pronounced in the control benchmark cheese and T3 cheese, which had a strong positive correlation, as shown in the following equations: C; y = 0.08x + 3.59 and T3; y = 0.0.06x + 3.74. T2 cheese coated with a PFUV did not correlate with the progress of the ripening period.

### 3.6. Acidity and pH Values of Ras Cheese

Throughout the ripening period, there were significant differences in acidity and pH values (*p* ˂ 0.05) in all Ras cheese treatments (Figure 2E,G). There was a lower acidity development and a slight increase in pH values in all fresh Ras cheeses and during the ripening period. It was noticed that the control cheese sample had the highest acidity at the beginning and during the ripening period. T2 cheese coated with a PFUV had the lowest acidity compared with other Ras cheese treatments, especially at the end of the ripening period. However, in the pH values of the experimental Ras cheese, it could be observed that T2 cheese coated with a PFUV had the highest pH value, while T1 cheese coated by paraffin wax had the lowest pH value when fresh compared with other Ras cheese treatments. The same approach was observed after a month of the ripening period.

Simple linear correlation (SLR), as shown in Figure 2F,H, was used to better understand the relationship between the acidity percentage and the pH value with the ripening period. Acidity % had a positive correlation with the ripening period in all Ras cheeses. Although, pH values had a negative correlation with the ripening period. The acidity% increased throughout the ripening period for all Ras cheese treatments and vice versa for pH values, as illustrated by the equations. Acidity correlation equations are as follows: C, y = 0.007x + 0.97; T1, y = 0.013x + 0.91; T2, y = 0.009x + 0.90; and T3, y = 0.011x + 0.92. T2 cheese coated with a PFUV had a more negative correlation compared with other Ras cheese treatments, as shown in the following pH equations: T2 y = −0.09x + 5.75, C, y = −0.07x+ 5.58; T1 y = −0.10x + 5.51; and T3 y = −0.05x + 5.56.

### 3.7. Sensory Evaluation of Ras Cheese

Data illustrated in Figure 3 indicated the sensory parameters of texture, color, and flavor, as well as the overall acceptability of cheese treatments that were affected by different kinds of coating materials during the ripening period. It could show there were no significant differences (*p* ˂ 0.05) throughout the ripening period in all Ras cheese treatments except in T2 cheese, which was coated with a PFUV and had the lowest texture (Figure 3A) compared with other Ras cheese treatments after 6 months of ripening. Although, texture scores increased in all Ras cheese treatments as the ripening period progressed. Additionally, there were no significant differences in color scores (Figure 3C) of all Ras cheese treatments throughout the ripening period. By prolonging the ripening period, the color scores of Ras cheese treatments increased in all Ras cheeses.

In addition, the control sample cheese had the lowest flavor score (Figure 3E) compared with other Ras cheese treatments after a month of ripening. As the ripening period increases, the flavor scores increase in all Ras cheese treatments. The results illustrated that there was a positive correlation between the sensory properties of Ras cheese treatments and the ripening period (Figure 3B,D,F,H). The control Ras cheese had a strong positive correlation with texture, as shown by the followung the equation: texture C; y = 0.13x + 2.41, while T3 cheese coated with a plastic film treated with natamycin had a strong positive correlation in color, flavor, and overall grade, as illustrated by the following equations: T3; y = 0.13x+ 2.66—T3; y = 0.17x + 2.64; and T3; y = 1.39x + 79.2, respectively.

### 3.8. Volatile Compounds of Ras Cheese

Volatile compounds of the Ras cheese treatments, which have the main effect on the cheese flavor, were identified (Figure S1). In the headspace of extracts, 36 volatile compounds were identified and classified into chemical groups such as esters, alcohols and fatty acids (Table S1). The results showed that at the exact retention time, different volatile compounds had corresponding concentrations expressed as a percentage of the peak area of the total separations. Volatile compounds were identified using purge-and-trap TDCT GC-MS. Many different compounds were detected and characterized in the cheese treatments.

Data in Figure 4 showed the volatile compounds of cheese. The control Ras cheese sample contains 42.07% esters, 1.82% alcohols, 54.44% fatty acids, and 1.68% other compounds. T1 Ras cheese coated with paraffin wax contains 7.82% esters, 1.30% alcohols, 27.41% fatty acids, and 63.74% other compounds. T2 Ras cheese, which is coated with a PFUV, contains 2.95% esters, 0.82% alcohols, 89.43% fatty acids, and 6.79% other compounds. T3 Ras cheese coated with a plastic film treated with natamycin contains 54.88% esters 0.33% alcohols, 44.87% fatty acids, and 0.00% other compounds.

Moreover, a two-way hierarchical cluster analysis (HCA) and its associated heat map using the abundances of volatile organic compounds (VOCs) of Ras cheese treated with different coating materials over the six-month ripening period is presented in Figure 4E. It could be indicated that T2 cheese coated with a PFUV has the greatest % of eicosanoic acid, hexadecanoic acid, 1(2-aminothoxy) hydroyphosphinyl) ethanediyl ester, pentadecanoic acid, and 1-(+)—ascorbyl palmitate 2, 6 dihexadecanoate. T3 cheese coated with a plastic film treated with natamycin has the highest % of n-Butyl ricinoleate and ascorbyl palmitate. In addition, T1 cheese has the highest % of glycerol 2-acetate 1, 3-dipalmiate. Data also revealed that the control sample cheese, T1, as well as T3 cheese, had the highest % of z-8-methyl-9-tetradecanoic acid compared with T2 cheese coated with a PFUV (Figure 4E).

Furthermore, Figure 5 presents the principal components analysis (PCA) and its associated biplot of volatile organic compounds (VOCs) of Ras cheese treated with different coating materials over a six-month ripening period. Ras cheese treatments were classified into four groups. All Ras cheese treatments were in a similar cluster in each region of the PCA and the cheeses varied pronouncedly. T1 and the control sample Ras cheese were in the same region as the PCA, as they contain the following compounds: 2-Pentanone, 4-hydroxy-4-methyl/Dodecanoic acid/Phenylacetic acid/Olic acid/Oxiranedodecanoic acid, 3-acetyl cis/Dodecyl cis-9,10-epoxy octadecanoate/9-Octadecenoic acid, 1,2,3-propanetriyl ester/Trans-13-Octadecanoic acid/Z-methyl-cis-7,8-epoxy mono decane/n-Butyl ricinoleate/9-Octadecanoic acid (Z)—2-butoxyethyl/Ethyl iso allocholate/Glycerol z-acetate 1,3-dipalmiate/olic acid eicosyl ester/Palmitic acid/Hexadecanoic acid, Z-(octadecyl) oxy) ethyl ester/Z-8-methyl-9-tetradecen-1-ol acetate/Z-8-methyl-9-tetradecenoic acid/2-Hexadecanol, and Triarachine.

## 4. Discussion

Cheese is a short-life product due to its dynamic changes in biochemical properties throughout processing, ripening, and marketing. So, the coating process has a vital role in the cheese industry [31]. The main purpose of coating cheese is to stop or reduce degradation processes that occur during the ripening period. Dehydration, oxidation, and preventing the development of an unacceptable off-flavor that takes place due to undesirable microorganisms or with other external populations, as well as decrease the rate of metabolic of ripening strains [32]. The coating process maintains ripened cheese from contaminated and undesirable changes during the ripening period, with a high loss of moisture content. The coating acts as a barrier that could decrease the loss in moisture content and enhance the sensory properties of cheese. Furthermore, coating cheese helps to lengthen the shelf-life of cheese during the ripening period by execrating its characteristics [33,34]. In addition to the progress of ripening period, the moisture content of all cheese samples significantly decreased, which could be due to the biochemical changes and lactic acid development, which cause curd contraction and expulsion of the aqueous phase of cheese [35].

Characteristics of coated Ras cheese were significantly affected by the coating technique. From our findings, the loss of moisture content from coated cheese during the ripening periods of T1, T2, and T3 indicated the permeability of water vapor, which is generally considered to be the ability of a coated film as a barrier for water vapor diffusion, which happened because of the hydrophilic portion of a coating film that is particularly important for coated cheese. Water vapor permeability of coated cheese during ripening is affected by different function factors, such as water content, the degree of porosity, and the composition of coating, as well as the ratio between hydrophilic and hydrophobic [36]. Matured coated Ras cheese with a plastic film treated with natamycin T3 had the lowest moisture at the end of the storage period compared with both uncoated and coated Ras cheese. The combination of the vacuum coating technique of the cheese with the ripening period under controlled conditions in T1 cheese led to limiting the proliferation of anaerobic pathogens that may be present in contaminated cheese. That effect could be due to the lower temperature during the ripening period, vacuum conditions, and LAB activity [37,38].

Furthermore, the results illustrated that the titratable acidity of all cheese treatments increased gradually at the end of the ripening period. The total acidity of the cheese is naturally caused by milk constituents, in addition to acidity, which developed during cheese ripening. This could be related to the degradation of intermediate compounds of protein and amino acids, as well as fatty acids resulting from fat hydrolysis [39]. The change in pH values of cheese throughout the ripening period of all Ras cheese could be related to the influence of the growth of starter lactic acid bacteria in pasteurized milk cheeses. Additionally, this could be attributed to the utilization of lactic acid, the formation of non-acidic decomposition products, and the liberation of alkaline products of protein decomposition [26].

We find that Ras cheese coated by the vacuum technique, T1, had different characteristics compared with another type of cheese, and this may be due to the vacuum technique, which may have some issues. Likewise, infiltration of the vacuum coating material happened due to the presence of lactate, calcium, and tyrosine crystals. Those crystals formed on the cheese surface cope with a long ripening time or storage period [39]. The reason for formatting these crystals on the cheese surface may be related to the loss of vacuum materials’ integrity. Furthermore, coated cheese with the vacuum technique may lead to a rise in its acidity during the ripening period due to whey residuals in the cheese, which approaches the cheese surface or an incision inside the cheese matrix that causes a rise in lactic acid concentration [40], which also may affect the profile of volatile compounds of coated cheese.

Additionally, the T3 coating of Ras cheese with a plastic film treated with natamycin did not have a negative or significant effect on its sensory characteristics when fresh or during the ripening period. That may be because natamycin affected microorganisms’ growth, which causes spoilage in stored cheese. However, natamycin did not affect the activity of LAB; thus, treated coating material with natamycin kept the sensory characteristics of coated cheese during ripening [41,42,43].

We observed that coated cheese with paraffin wax, T1, had the highest moisture during the ripening period, which may be due to paraffin wax having good characteristics and a moisture barrier that decreased moisture loss [44]. Additionally, paraffin wax that was used in cheese coating affected the flavor profile of coated cheese, which may be due to the migration of paraffin wax inside the cheese through the wax layer during the ripening period [45,46]. Additionally, paraffin wax is one of the lipid-based coating categories that had a highly satisfactory moisture barrier during use, and it usually forms relatively low elastic surfaces [47].

The flavor change in cheese during ripening is a complex process when the catabolic pathways are involved. The cheese curd produces different flavor compounds, which lead to discrimination of maturated cheese throughout ripening. The major biochemical pathways that occurred throughout the ripening of cheese involved the liberation of free fatty acids, the degradation of the casein matrix to peptides and free amino acids associated catabolic reactions, the reactions of catabolism of free fatty acids, and the metabolism of lactate and citrate in cheese [48].

The flavor profile characteristics of matured Ras cheeses are affected by the proteolysis of casein lipolysis [49]. The typical cheese flavor results from further degradation of amino acids because of the pathways of starter bacteria that metamorphosize amino acids [50]. Based on sensory evaluation and chemical analysis of cheeses, various groups of volatile compounds have been identified as being responsible for the final aroma of cheese. These compounds comprise alcohols, aldehydes, fatty acids, and other compounds [51] [29].

The flavor of the cheese is produced from the degradation of milk protein, lactose, fat, and citrate because of the enzymes from milk, microorganisms, and rennet throughout the ripening period of cheese [52]. The process of proteolysis of casein is considered the main factor that is helpful in the development of the flavor of ripened cheese during ripening. The result of the proteolysis of casein is unlimited amino acids that are considered precursors in matured cheese, and the formation of amino acids is important for the development of cheese’s flavor [53,54].

The accumulation of volatile compounds in matured cheese during the ripening period could be due to different metabolic pathways such as proteolysis, lactose hydrolysis, and lipolysis [55,56]. The LAB, salt, and ripening conditions are factors that are responsible for the evolution of volatile compounds in cheese [57]. The types of fatty acids, alcohols, and other volatile compounds in cheese affected the flavor extensity of ripened cheese, and this may be due to the difference in microflora and its active enzymes in cheese [58]. In addition, the heat treatment, as well as different coating techniques and materials, are responsible for the type and concentration of fatty acids and their drivers in ripened cheese [59]. The un-coated control cheese (as a benchmark cheese) had a different percentage of volatile compounds compared to other excremental-coated cheeses, and this could be due to the presence of undesirable microflora, which degraded cheese components at various levels. Additionally, the limitation of aeration in coated cheese affects the volatile compounds of cheese. The coating of cheese reduces the oxygen and pressure in cheese [60].

By applying coating films in T1, T2, and T3 cheese on the cheese surface moisture, condensation/migration could be controlled. The exchange of natural vaporable flavor and color compounds between cheese and its environmental surroundings could be restricted, as the coating acts as a gas barrier [61,62]. The vacuum coating technique used in T2 cheese depends on getting rid of the air around the cheese with a coating material that modifies the inner atmosphere of the cheese coat that does not allow permeability of O_2_ and other gases. In this process, the coated cheese difference between the inner pressure, which is much lower, and the atmospheric pressure outside the cheese. So, the amount of oxygen inside the coated cheese became less than 1%, which may influence the sensory properties of the coated cheese [63].

Cheese did not have significant differences in salt content. This may be due to the way of salting cheese in a brine solution without dry salting. That method of salting did not produce a change in the salt content of cheese compared with the dry salting method, which exhibited significantly lower characteristics of cheese [64].

## 5. Conclusions

This study investigated the changes in sensory properties, physico-chemical characteristics, and aromas of Ras cheese under different coating techniques including paraffin wax, the plastic film under a vacuum (PFUV), the plastic film treated with natamycin, and non-coated cheese. Although all tested coating techniques slightly changed the characteristics of Ras cheese, coating Ras cheese using the PFUV significantly enhanced the total nitrogen content over a 6-month ripening period. Furthermore, our findings showed that coating Ras cheese using the PFUV or a plastic film treated with natamycin had better characteristics in the physio-chemical and sensory properties, as well as aroma-related volatile compounds. It is worth mentioning that none of the tested coating techniques changed the salt consumption of coated cheese compared with the non-coated control. Additionally, coating using the PFUV might play a key role in extending the shelf-life of Ras cheese without changing its sensory properties. The usage of the PFUV might be promising due to its excellent barrier characteristics against loss of moisture, as well as its low cost. These properties made it highly convenient for use. The new way of coating did not cause any negative characteristics or off-flavor compounds. This coating technique could be used with other types of cheese, such as semi-hard cheese.

## Data Availability

All data generated or analyzed in this study is included in this published article.

## Supplementary Materials

The following supporting information can be downloaded at https://www.mdpi.com/article/10.3390/foods12102023/s1, Table S1. Different volatile organic compounds (VOCs) of Ras cheese treated with different coating materials over a six-month ripening period; Figure S1. Representative chromatograms of different volatile organic compounds (VOCs) of Ras cheese treated with different coating materials over a six-month ripening period.
