# Supplementary material for: Changes in Sensory Properties, Physico-Chemical Characteristics, and Aromas of Ras Cheese under Different Coating Techniques
Source: Foods. 2023 May 17;12(10):2023. doi: 10.3390/foods12102023 (PMC10217755; doi:10.3390/foods12102023)
Supplement: Supplementary file 1 [file foods-12-02023-s001.zip › foods-2351269-supplementary.pdf]

foods

Supplementary Materials

Changes in Sensory Properties, Physico-Chemical Characteristics, and Aromas of Ras cheese Under Different Coating Techniques

Dina A. Amer [1,*], Abdinn A. M. Albadri [2], Hanaa A. El-Hamshary [1], Yasser Nehela [3,4,*], Abeer H. Makhlouf [5], Mohamed Y. El-Hawary [1] and Sameh A. Awad [6]

[1] Department of Food Science and Technology, Faculty of Agriculture, Tanta University, Tanta 31527, Egypt
[2] Department of Biology, College of Science, King Khalid University, Abha 62529, Saudi Arabia; abdin@kku.edu.sa
[3] Department of Agricultural Botany, Faculty of Agriculture, Tanta University, Tanta 31527, Egypt
[4] Department of Plant Pathology, Citrus Research and Education Center, University of Florida, Lake Alfred, FL 33850, USA
[5] Department of Agricultural Botany, Faculty of Agriculture, Minufiya University, Shibin El-Kom 32511, Egypt
[6] Dairy Microorganisms and Cheese Research Laboratory (DMCR), Department of Dairy Science and Technology, Faculty of Agriculture, Alexandria University, Alexandria 21545, Egypt; sameh.awad@alexu.edu.eg
* Correspondence: dina.amer@agr.tanta.edu.eg (D.A.A.); yasser.nehela@agr.tanta.edu.eg (Y.N.)

Table S1. Peak areas and percentages of different volatile organic compounds (VOCs) of Ras cheese treated with different coating materials over a six-month ripening period.

RT	Compound	Control		T1		T2		T3	
		Area	%	Area	%	Area	%	Area	%
5.21	2-Pentanone, 4-hydroxy-4-methyl-	1.37	1.82	1.75	0.34	0.81	0.14	1.12	0.20
19.45	n- Decanoic acid	1.15	1.53	3.80	0.73	-	-	0.66	0.12
24.10	Dodecanoic acid	2.07	2.75	3.51	0.67	0.79	0.14	1.25	0.22
28.54	Tridecanoic acid	-	-	9.28	1.78	-	-	3.16	0.55
30.48	Phenylacetic acid	1.61	2.15	1.70	0.33	-	-	-	-
31.15	Pentadecanoic acid	-	-	-	-	12.77	2.23	0.94	0.16
32.26	Palmitic acid	1.41	1.88	39.35	7.55	1.17	0.20	-	-
32.64	Myristic acid	-	-	-	-	-	-	0.80	0.14
33.58	Estra-1,3,5(10)-trien-17á-ol	-	-	-	-	-	-	0.39	0.07
34.17	9- Octadecanoic acid (Z) methyl ester	1.83	2.44	2.07	0.40	1.57	0.27	1.12	0.20
34.06	Eicosanoic acid	-	-	-	-	-	-	1.97	0.34
35.09	Olic acid	21.94	29.24	29.81	5.72	-	-	26.20	4.59
35.92	Z-8-methyl-9-tetradecanoic acid	-	-	13.37	2.57	2.25	0.39	26.20	4.59
36.50	Trans-13-octadecanoic acid	0.64	0.85	-	-	-	-	-	-
36.92	Hexadecanoic acid, 1-(hydroxymethyl)-1,2-ethanediyl ester	-	-	-	-	1.07	0.19	7.05	1.23
37.21	Diethylene glycol mono laurate	0.00	0.00	0.00	0.00	3.77	0.66	7.34	1.28
38.06	l-(+)-Ascorbic acid 2,6-dihexadecanoate	3.46	4.61	4.30	0.83	13.20	2.30	0.95	0.17
38.43	2-Hexadecanol	-	-	5.04	0.97	-	-	0.39	0.07
39.49	E-8-methyl-9-teradecen-1-ol acetate	0.64	0.86	7.86	1.51	1.36	0.24	-	-
39.72	Oxiranedodecanoic acid, 3- octyl cis	9.19	12.25	1.81	0.35	-	-	-	-
40.10	Dodecyl cis-9,10-epoxy octadecanoate	24.04	32.04	3.88	0.75	-	-	-	-
40.25	10-undecenoic acid	-	-	-	-	1.71	0.30	0.52	0.09
40.79	Hexadecanoic acid, Z-(octadecyl) oxy) ethyl ester	-	-	16.15	3.10	1.37	0.24	-	-
41.16	Z-methyl-cis -7,8- epoxy mono decane	1.73	2.31	6.20	1.19	-	-	-	-
41.46	Octadecanoic acid 2,3- bis (1- oxotetradecyl) oxy) propyl ester	-	-	7.04	1.35	7.32	1.28	-	-
41.79	Hexadecanoic acid, 1(2-aminothoxy) hydroryphosphinyl) ethanediyl ester	-	-	-	-	119.23	20.77	-	-
42.54	9-Octadecenoic acid, 1,2,3 -propanetriyl ester, (E,E,E)	1.01	1.34	-	-	-	-	-	-
42.90	Triarachine	1.26	1.68	32.78	6.29	-	-	-	-
43.45	9-Octadecenoic acid (Z)- ,2-butoxyethyl ester	-	-	14.88	2.86	-	-	-	-
43.71	Ethyl iso-allocholate	-	-	10.42	2.00	-	-	-	-
44.47	n-Butyl ricinoleate	1.68	2.24	8.07	1.55	-	-	305.17	53.43
44.53	Olic acid ,eicosyl ester	-	-	2.94	0.56	-	-	-	-
44.67	Eicosanoic acid, 2-(1-oxoy) 1-(1-oxohexadecyl)oxy)methyl) ethyl ester	-	-	-	-	390.46	68.03	-	-
45.33	Ascorbyl palmitate+C20	-	-	-	-	15.05	2.62	185.91	32.55
45.72	Glycerol 2-acetate 1,3- dipalmiate	-	-	294.93	56.61	-	-	-	-

Control: uncoated Ras cheese (the benchmark control); T1: Ras cheese coated with paraffin wax; T2: Ras cheese coated with a plastic film under a vacuum (PFUV); and T3: Ras cheese coated with a plastic film treated with natamycin.

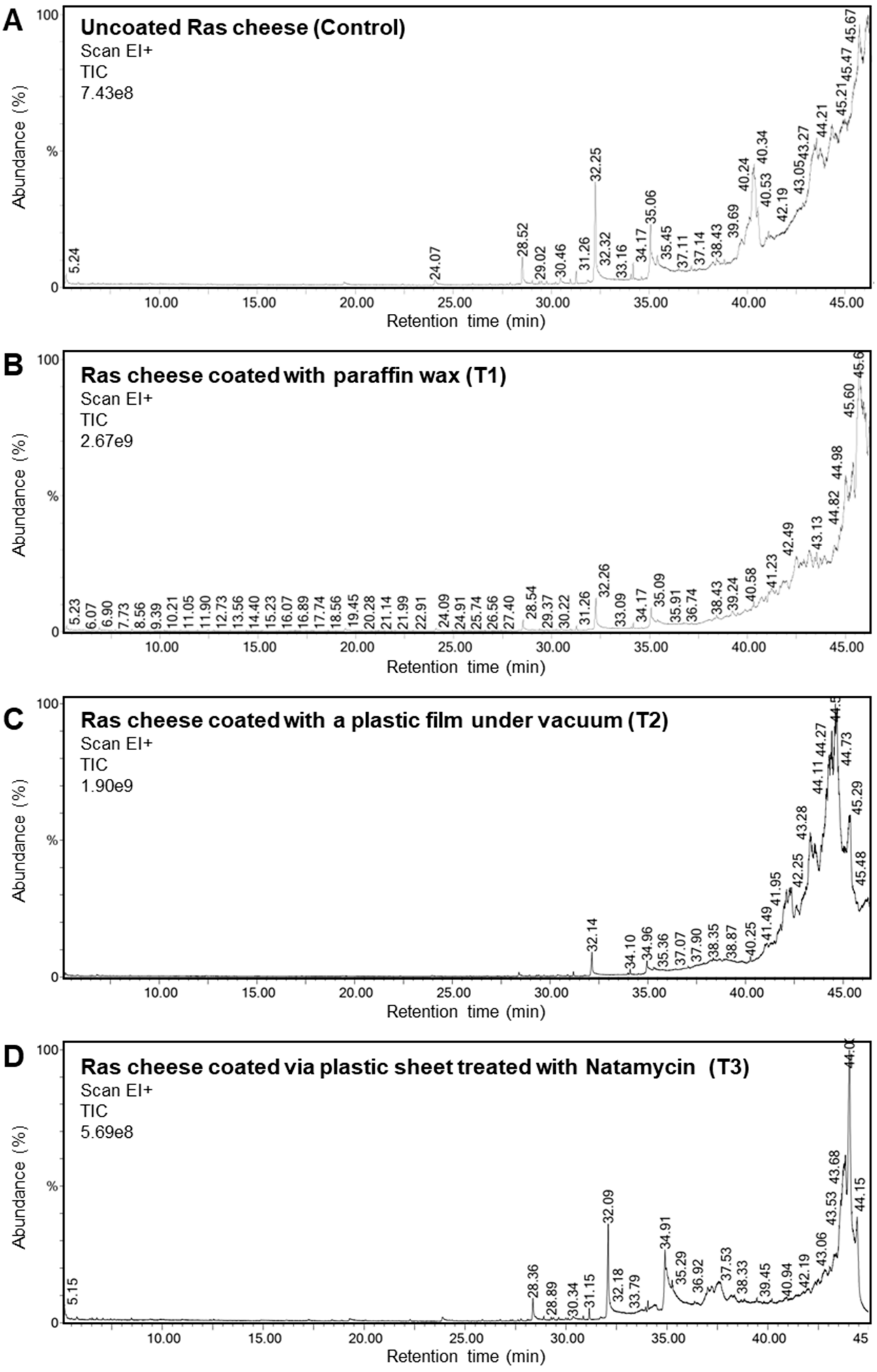

Figure S1. Representative chromatograms of different volatile organic compounds (VOCs) of Ras cheese treated with different coating materials over a six-month ripening period. (A) Representative chromatogram of uncoated Ras cheese (the benchmark control), (B) representative chromatogram of Ras cheese coated with paraffin wax (T1), (C) representative chromatogram of Ras cheese coated with a plastic film under a vacuum (PFUV; T2), and (D) representative chromatogram of Ras cheese coated with a plastic film treated with natamycin (T3).
